# Association between a western diet and asthma among children and adolescents

**DOI:** 10.1038/s41598-024-64008-5

**Published:** 2024-06-09

**Authors:** Arezoo Sadat Emrani, Bahareh Sasanfar, Mohammad-Reza Jowshan, Nasrin Behniafard, Zahra Nafei, Amin Salehi-Abargouei

**Affiliations:** 1 Research Center for Food Hygiene and Safety, School of Public Health, Shahid Sadoughi University of Medical Sciences, Yazd, Iran; 2 Yazd Cardiovascular Research Center, Non-Communicable Diseases Research Institute, Shahid Sadoughi University of Medical Sciences, Yazd, Iran; 3 Department of Nutrition, School of Public Health, Shahid Sadoughi University of Medical Sciences, Yazd, Iran; 4 Children Growth Disorder Research Center, Shahid Sadoughi Hospital, Shahid Sadoughi University of Medical Sciences, Ebne sina boulevard, Yazd, Iran; 5 Department of Allergy and Clinical Immunology, Shahid Sadoughi University of Medical Sciences, Yazd, Iran; 6 Cancer Research Center, Cancer Institute of Iran, Tehran University of Medical Sciences, Tehran, Iran; 7 Department of Clinical Nutrition, School of Nutritional Sciences and Dietetics, Tehran University of Medical Sciences, Tehran, Iran; 8 Student Research Committee, Shahid Sadoughi University of Medical Sciences, Yazd, Iran

**Keywords:** Western diet, Asthma, Cross sectional, Children, Western pattern, Immunology, Diseases, Nutrition, Paediatrics

## Abstract

Several risk factors including environmental exposures, socioeconomic status, and dietary factors including dietary patterns have been considered for childhood Asthma. The present study tried to examine the association between a western-style pattern and the likelihood of asthma and its symptoms in Yazd, Iran. In the present cross-sectional study, dietary intakes of elementary and high-school children were obtained through a validated GAN questionnaire. The GAN questionnaire, derived from the ISAAC questionnaire was used to assess the symptoms of allergic diseases and their related risk factors. A western dietary pattern score considered 9 food groups including chicken eggs, margarine, butter, sugar, fast foods, soft drinks, snacks, sauce, and chocolate. In total 7667 children aged 10.9 ± 3.35 years were included in the current investigation. Boys with higher adherence to western dietary pattern had a higher risk of wheezing in the past 12 months (OR 1.37, 5% CI 1.01–1.87, *P* = 0.04) and this association was also observed in the whole population (OR 1.30, 5% CI 1.05–1.60, *P* = 0.01). However, after adjustment for confounders this relation did not remain significant in boys. Our results support the hypothesis that a western dietary pattern is associated with an increased risk of wheezing in the past 12 months in children with asthma. Future prospective studies are needed to confirm this finding.

## Introduction

Asthma is a chronic inflammatory disease^1^ that might have genetic and environmental background^2^. As a global concern, the burden of asthma on patients and the health care system of governments around the world is increasing and about 15 million disability-adjusted life years lost annually are due to asthma^3^. Approximately, 300 million people are affected by asthma, and statistics show that this number is rising^4^. Although the highest prevalence of asthma is in high-income countries, low-income countries are also affected by the disease^5^. The prevalence of this respiratory disease in children was reported 10.1% in Brazil^6^, 5.35% in India^7^, 10.6% in Oman^8^ and 6% in Iran^9^.

Childhood asthma might have a variety of risk factors, including no breastfeeding, family structure, socio-economic status, infections, and environmental exposures^10^. Dietary intake is another important factor in the prevention and management of the disease^11^. Several studies have examined the effect of different dietary patterns on asthma^12–14^. Papamichael et al. conducted a systematic review to investigate the efficacy of a Mediterranean-style dietary pattern on childhood asthma and they found that adherence to this dietary pattern could be inversely associated with the disease^12^. A cross-sectional study suggested that a dietary pattern with a high intake of fat and sugar and a low intake of rice, fruits, and vegetables may increase the risk of asthma in children^13^. A case–control in Ian was conducted to determine dietary patterns in asthmatic patients^15^. They observed that people with asthma probably consume less fast food, salty foods and meats to control the symptoms of this disease^15^. In addition, increasing urbanization along with changes in dietary patterns might affect the risk of asthma, which makes diet more important^16^. Moreover, changes in diets associated with Westernization might contribute to its burden^17^.

Western dietary pattern is high in processed foods, refined grains, high fat dairy, and sugary products^18^ and low in fruits, vegetables and whole grains^19^. In a study by Laurent Guilleminault et al.^20^ the relationship between diet and asthma was examined, and they found a direct association between a Western dietary pattern and risk of asthma. However, a review study found no association between a western diet and the incidence of asthma in adults, but it did show a possible link between this dietary pattern and asthma morbidity^18^. Results of the Nurses’ Health Study also showed that there is no relationship between a western dietary pattern and the risk of asthma^21^. It seems that the western diet is a pro-inflammatory diet^22^ and adherence to this dietary pattern may increase inflammatory markers such as hs-CRP and IL-6^23^. It has been shown that the consumption of a pro-inflammatory diet may worsen asthma conditions^20,24^. In addition, the western diet may indirectly affect asthma. This dietary pattern plays a key role in the obesity epidemic^25^ and overweight and obese people have a higher risk for developing asthma^26^.

Studies have shown that dietary patterns in the Middle East have changed toward consuming foods with higher calories and more sugar^27^. Also, the western diet has become very common in this region^19^. To the best of our knowledge, no study has been conducted in the Middle East to investigate the relationship between a western dietary pattern and childhood asthma. Therefore, the current study aimed to assess the association between adherence to a western-style dietary pattern and asthma among children and adolescents.

## Methods

### Participants

This cross-sectional study was performed in early 2020 in Yazd, Iran. It was conducted as part of the Global Asthma Network (GAN) which is a cross-sectional, multi-country, multi-center, epidemiological research that follows and expands the methodology of ISAAC Phase three^28^. According to the GAN protocol, a minimum sample size of 3000 participants were required to determine the prevalence of asthma^29^. In the present study, random sampling was performed from 36 elementary and 48 high schools (state and private) in 2 educational districts of Yazd using a cluster sampling design. Non-Iranian participants were excluded. Since our data collection coincided with Covid-19 quarantine, all students aged 13–14 and parents of students aged 6–7 were asked to complete online electronic questionnaires about asthma and its symptoms and risk factors which were sent to the virtual education groups of schools. Some of our data were collected before the Covid-19 pandemic through a paper questionnaire. In total 7214 children (6–7 year) and 3026 adolescents (13–14 years) participated in our study and completed the questionnaire with 71.3% and 83.5% response rates, respectively. Demographic data that appeared to be incorrect was then re-checked and corrected as needed. 

The GNA study in Iranian children is ethically approved by the ethics committee of Shahid Sadoughi University (SSU) of Medical Science Yazd, Iran (IR.SSU.REC.1398.244). The cuurrent study was also ethically approved by this committee (ethics code: IR.SSU.SPH.REC.1400.137). Conducing the study in schools was permitted by the Yazd educational administration. Written informed consent was obtained from all subjects/patients.

### Asthma and its symptoms confirmation

The GAN questionnaire which was derived from the ISAAC questionnaire^30^ was used for the current investigation. The questionnaire includes questions about the symptoms of allergic diseases and their related risk factors. First the English version of questionnaire was translated into Persian and to appraise it, a pilot study with 100 selected subjects was conducted. Cronbach's alpha was used to confirm the reliability of this version of questionnaire and the alpha coefficient for asthma symptoms was estimated to be 0.862, which indicates appropriate internal consistency. Then, the questionnaire was translated back into English again and sent to the GAN principals in New Zealand for approval. For the present study, there were some questions about asthma symptoms, "use of asthma medication" and "asthma confirmed by a doctor". Current asthma was defined as history of confirmed asthma by a doctor and having had wheezing and/or use of asthma medication in the past 12 months.

### Assessment of dietary intakes

In our study, GAN questionnaire which was containing 26 food groups on usual dietary intake of children, during the last 12 months^31^ was used. The frequency of food consumption was assessed by three options in the answer section (never or occasionally/once or twice a week/most or all days of week).

### Assessment of Western dietary pattern score

In the current study, based on previous studies 9 food groups through data of food frequency questionnaire were selected to assess the Western diet score^32^: chicken eggs, margarine, butter, sugar, fast foods, soft drinks, snacks, sauce, and chocolate. A score of 1–3 was assigned for each item, based on the frequency responses for each food item (never, weekly, every day). The overall score of each participant was obtained by summing the score of each food item, which ranged between 9 and 27. Finally, participants were categorized into tertiles based on their scores.

### Assessment of other variables

A self-reported online GAN questionnaire was used to collect data on participants’ height, weight and ethnicity (Kord, Turk, Persian, Lor, Arab, Balooch) and also other variables including watching television and computer use (2–4 h/5–8 h/9–14 h a day). Moreover, by using children’s weight (kg) and height (m) the body mass index (BMI, kg/m^2^) was calculated.

### Statistical analysis

All analyzes were performed by using STATA software version 14 (State Corp., College Station, TX). To compare ordinal qualitative variables and continuous variables in children with or without Asthma confirmed by a doctor, chi-square test and independent sample t-test were used, respectively. Logistic regression was used to assess the association between western dietary pattern intake and asthma confirmed by a doctor, current asthma, use of asthma medication and wheezing in the past 12 months in crude and multi-variable adjusted models. Adjustment was done for age and sex, watching TV and computer use and the BMI. A *P*-value less than 0.05 was considered as statistically significant.

### Ethical standards disclosure

This study was conducted according to the guidelines laid down in the Declaration of Helsinki and all procedures involving human subjects/patients were approved by the Shahid Sadoughi University (SSU) of Medical Science Yazd, Iran (IR.SSU.REC.1398.244). Also, the current study was ethically approved by the ethics committee of (ethics code: IR.SSU.SPH.REC.1400.137).

## Results

In total 7667 participants were included in the current study. The general characteristics of the participants according to asthma confirmed by a doctor and medication prescribed asthma are presented in Table 1. Of the 324 participants with asthma confirmed by a doctor, 58.02% were boys. Boys with medication prescribed asthma, were 61.7%. Children with doctor diagnosed (*P* < 0.001) and medication prescribed asthma were older (*P* = 0.05) than children without asthma. Frequency of ethnicity were different between those with and without medication prescribed asthma (*P* < 0.01). Ever had wheezing and wheezing in the past 12 months were significantly lower in children with doctor diagnosed compared to those without these conditions and were significantly different among medication prescribed asthma (*P* < 0.001).

**Table 1:** General characteristics of the subjects according to asthma confirmed by a doctor.

Variables	Doctor diagnosed asthma		*P*-value	Medication prescribed asthma		*P*-value^a^
	Without (n = 7343)	With (n = 324)		Without (n = 7476)	With (n = 191)	
Sex						
Male	3226 (43.93)	188 (58.02)	0.000	3296 (44.09)	118 (61.7)	0.000
Female	4117 (56.07)	136 (41.98)		4180 (55.9)	73 (38.2)	
Age (years)	10.9 ± 3.37	11.7 ± 2.94	0.000	10.9 ± 3.36	11.3 ± 3.16	0.05
BMI (kg/m^2)^	18.9 ± 10.4	19.1 ± 4.18	0.35	18.97 ± 10.3	18.73 ± 3.97	0.37
Ethnicity			0.13			0.003
Kord	38 (0.52)	5 (1.54)		38 (0.51)	5 (2.62)	
Turk	73 (0.99)	2 (0.62)		74 (0.99)	1 (0.52)	
Persian	7064 (96.2)	311 (95.9)		7192 (96.2)	183 (95.8)	
Lor	62 (0.84)	4 (1.2)		64 (0.86)	2 (1.05)	
Arab	55 (0.75)	1 (0.31)		56 (0.75)	0 (0.0)	
Baloch	51 (0.69)	1 (0.31)		52 (0.70)	0 (0.0)	
Physical activity (watch TV and computer use)			0.38			0.29
2–4 h	3945 (53.7)	163 (50.3)		4016 (53.7)	92 (48.1)	
5–8 h	2463 (33.5)	103 (34.8)		2506 (33.5)	70 (36.6)	
9–14 h	935 (12.7)	48 (14.8)		954 (12.7)	29 (15.1)	
Ever had wheezing						
Yes	1160 (15.8)	96 (29.6)	0.000	1109 (14.8)	147 (76.9)	0.000
No	6183 (84.2)	228 (70.3)		6367 (85.1)	44 (23.0)	
Wheezing (in the past 12 months )						
Yes	553 (7.5)	56 (17.2)	0.000	518 (6.9)	91 (47.6)	0.000
No	6790 (92.4)	268 (82.7)		6958 (93.0)	100 (52.3)	

Values are mean (SD) or percentages.

^a^χ^2^ Test for ordinal qualitative variables and t-test for continuous variables.

Nine food items were selected to assess western dietary pattern score. The frequency of fast foods (*P* = 0.03), soft drinks (*P* = 0.004) and sauce (*P* = 0.01) intake was significantly higher in children with doctor diagnosed asthma compared to those without the disease. Also, children with use of asthma medication compared with those without it had higher frequency intake of margarine (0.05), fast foods (*P* = 0.03) and sauce (*P* = 0.01) (Table 2).

**Table2:** Daily food of subjects according to asthma confirmed by a doctor and Use of asthma medication.

Variables	Asthma confirmed by a doctor		*P*-value	Use of asthma medication		*P*-value
	Without (n = 7343)	With (n = 324)		Without (n = 7476)	With (n = 191)	
Margarine						
Never	5434 (74.0)	238 (73.4)	0.12	5539 (74.0)	133 (69.6)	0.05
Weekly	1563 (21.2)	63 (19.4)		1584 (21.1)	42 (21.9)	
E very day	346 (4.7)	23 (7.1)		353 (4.7)	16 (8.3)	
Animal butter						
Never	3750 (51.0)	169 (52.1)	0.66	3826 (51.1)	93 (48.6)	0.43
Weekly	2768 (37.7)	115 (35.4)		2812 (37.6)	71 (37.1)	
Every day	825 (11.2)	40 (12.3)		838 (11.2)	27 (14.1)	
Eggs						
Never	657 (8.9)	24 (7.4)	0.56	666 (8.9)	15 (7.8)	0.2
Weekly	4612 (62.8)	203 (62.6)		4704 (62.9)	111 (58.1)	
Every day	2074 (28.2)	97 (29.9)		2106 (28.1)	65 (34.0)	
Fast food						
Never	4148 (56.4)	160 (49.3)	0.03	4217 (56.4)	91 (47.6)	0.03
Weekly	2931 (39.9)	152 (46.9)		2989 (39.9)	94 (49.2)	
Every day	264 (3.6)	12.(3.7)		270 (3.6)	6 (3.1)	
Sugar						
Never	1693 (23.0)	70 (21.6)	0.60	1717 (22.9)	46 (24.0)	0.86
Weekly	4065 (55.3)	177 (54.6)		4140 (55.3)	102 (53.4)	
Every day	1585 (21.5)	77 (23.7)		1619 (21.6)	43 (22.5)	
Soft drink						
Never	4105 (55.9)	151 (46.6)	0.004	4161 (55.6)	95 (49.7)	0.2
Weekly	2612 (35.5)	140 (43.2)		2672 (35.7)	80 (41.8)	
Every day	626 (8.5)	33 (10.1)		643 (8.6)	16 (8.3)	
Snacks						
Never	3762 (51.2)	159 (49.0)	0.25	3817 (51.0)	104 (54.4)	0.37
Weekly	3073 (41.8)	135 (41.6)		3137 (41.9)	71 (37.1)	
Every day	508 (6.9)	30 (9.2)		522 (6.9)	16 (8.3)	
Chocolate						
Never	3760 (51.2)	166 (51.2)	0.95	3828 (51.2)	98 (51.3)	0.95
Weekly	3006 (40.9)	134 (41.3)		3063 (40.9)	77 (40.3)	
Every day	577 (7.8)	24 (7.4)		585 (7.8)	16 (8.3)	
Sauces						
Never	3195 (43.5)	114 (35.1)	0.01	3243 (43.3)	66 (34.5)	0.01
Weekly	3482 (47.4)	175 (54.0)		3558 (47.5)	99 (51.8)	
Every day	666 (9.0)	35 (10.8)		675 (9.0)	26 (13.6)	

Values are percentages.

The association between adherence to western dietary pattern and asthma confirmed by a doctor is shown in Table 3. There was a decreasing trend but not significant association between adherence to a western dietary pattern and asthma confirmed by a doctor in girls, boys and the whole population.

**Table 3:** Association between adherence to western dietary pattern and Asthma confirmed by a doctor.

	Tertiles of western dietary pattern			
	T1	T2	T3	P_trend_
	0R (95% CI)	0R (95% CI)	0R (95% CI)	
Boys				
No. of With/without asthma	47/1069	60/1242	43/953	
Crude	1.00	1.09 (0.74–1.62)	1.02 (0.67–1.56)	0.89
Model 1	1.00	1.09 (0.73–1.61)	1.00 (0.65–1.53)	0.98
Model 2	1.00	1.06 (0.72–1.58)	0.93 (0.6–1.44)	0.78
Model 3	1.00	1.06 (0.72–1.57)	0.93 (0.6–1.44)	0.78
Girls				
No. of With/without asthma	68/1543	62/1412	44/1124	
Crude	1.00	0.99 (0.7–1.41)	0.88 (0.6–1.30)	0.57
Model 1	1.00	0.97 (0.68–1.39)	0.83 (0.56–1.22)	0.36
Model 2	1.00	0.98 (0.69–1.40)	0.84 (0.57–1.25)	0.42
Model 3	1.00	0.99 (0.69–1.41)	0.84 (0.56–1.24)	0.41
Whole population				
No. of With/without asthma	115/2612	122/2654	87/2077	
Crude	1.00	1.04 (0.8–1.35)	0.95 (0.71–1.26)	0.76
Model 1	1.00	1.02 (0.79–1.33)	0.9 (0.68–1.20)	0.52
Model 2	1.00	1.02 (0.78–1.32)	0.88 (0.66–1.18)	0.44
Model 3	1.00	1.01 (0.78–1.32)	0.88 (0.66–1.18)	0.44

Model 1: adjusted for age and sex (for total participants).

Model 2: further adjusted for watch TV & computer use.

Model 3: additionally adjustment for BMI.

No association was found between adherence to a western dietary pattern and the odds of current asthma in girls (OR 0.69, 95% CI 0.2–32) and the whole population (OR 0.51, 95% CI 0.24–1.07, *P* = 0.08). However, a significant negative trend in this relation was observed in boys’ population (OR 0.41, 95% CI 0.16–1.04, *P* = 0.04) (Table 4). Even after adjusting for BMI, watching TV and using of computer, this trend was remained significant (OR 0.39, 95% CI 0.15–1.01, *P* = 0.04).

**Table 4:** Association between adherence to western dietary pattern and current asthma.

	Tertiles of western dietary pattern			
	T1	T2	T3	P _trend_
	0R (95% CI)	0R (95% CI)	0R (95% CI)	
Boys				
No. of with/without current asthma	17/1005	12/1141	6/862	
Crude	1.00	0.62 (0.29–1.30)	0.41 (0.16–1.04)	**0.04**
Model 1	1.00	0.62 (0.29–1.31)	0.43 (0.16–1.10)	0.06
Model 2	1.00	0.6 (0.28–1.27)	0.39 (0.15–1.01)	**0.04**
Model 3	1.00	0.6 (0.28–1.26)	0.39 (0.15–1.01)	**0.04**
Girls				
No. of With/without current asthma	8/1439	9/1313	4/1030	
Crude	1.00	1.23 (0.47–3.20)	0.69 (0.2–2.32)	0.63
Model 1	1.00	1.26 (0.48–3.28)	0.75 (0.22–2.50)	0.73
Model 2	1.00	1.32 (0.50–3.45)	0.83 (0.24–2.79)	0.87
Model 3	1.00	1.32 (0.50–3.45)	0.83 (0.24–2.81)	0.88
Whole population				
No. of With/without current asthma	25/2444	21/2454	10/1892	
Crude	1.00	0.83 (0.46–1.49)	0.51 (0.24–1.07)	0.08
Model 1	1.00	0.81 (0.45–1.45)	0.53 (0.25–1.10)	0.09
Model 2	1.00	0.8 (0.44–1.45)	0.52 (0.24–1.10)	0.08
Model 3	1.00	0.8 (0.44–1.45)	0.52 (0.24–1.10)	0.08

Model 1: adjusted for age and sex (for total participants).

Model 2: further adjusted for watch TV and computer use.

Model 3: additionally adjustment for BMI.

Significant values are in [bold].

Adherence to western dietary pattern was not significantly related to using asthma medications in the whole population (OR 1.19, 95% CI 0.81–1.75, *P* = 0.35, Table 5) and subgroup analysis by sex. In boys (OR 1.37, 95% CI 1.01–1.87, *P* = 0.04) and the whole population (OR 1.30, 95% CI 1.05–1.60, *P* = 0.01), participants in the higher tertile of adherence to western dietary pattern had higher risk of wheezing in the past 12 months compared to those who were in the lower tertile (Table 6). Also, after adjusting for age and sex, this relationship was remained significant in the whole population and children who were in the top tertile of adherence to western dietary pattern had 24% chance for developing wheezing in the past 12 months than those in lower tertile (OR 1.24, 95% CI 1.00–1.53, *P* = 0.04), but in boys after adjustment for age and sex, disappeared.

**Table 5:** Association between adherence to western dietary pattern and Use of asthma medication.

	Tertiles of western dietary pattern			
	T1	T2	T3	P _trend_
	0R (95% CI)	0R (95% CI)	0R (95% CI)	
Boys				
No. of With/without asthma medication	35/1081	46/1256	37/959	
Crude	1.00	1.13 (0.72–1.76)	1.19 (0.74–1.90)	0.46
Model 1	1.00	1.12 (0.71–1.75)	1.15 (0.71–1.84)	0.55
Model 2	1.00	1.09 (0.7–1.72)	1.07 (0.66–1.74)	0.75
Model 3	1.00	1.09 (0.70–1.72)	1.08 (0.66–1.74)	0.75
Girls				
No. of With/without asthma medication	19/1592	35/1439	19/1149	
Crude	1.00	2.03 (1.16–3.57)	1.38 (0.73–2.62)	0.26
Model 1	1.00	2.03 (1.15–3.56)	1.36 (0.72–2.60)	0.28
Model 2	1.00	2.02 (1.15–3.56)	1.36 (0.71–2.60)	0.29
Model 3	1.00	2.01 (1.14–3.53)	1.36 (0.71–2.62)	0.28
Whole population				
No. of With/without asthma medication	54/2673	81/2695	56/2108	
Crude	1.00	1.48 (1.05–2.10)	1.31 (0.9–1.91)	0.14
Model 1	1.00	1.41 (1.00–2.01)	1.23 (0.84–1.80)	0.25
Model 2	1.00	1.39 (0.98–1.98)	1.18 (0.80–1.74)	0.36
Model 3	1.00	1.39 (0.98–1.98)	1.19 (0.81–1.75)	0.35

Model 1: adjusted for age and sex (for total participants).

Model 2: further adjusted for watch TV and computer use.

Model 3: additionally adjustment for BMI.

**Table 6:** Association between adherence to western dietary pattern and wheezing in the past 12 months.

	Tertiles of western dietary pattern			
	T1	T2	T3	P _trend_
	0R (95% CI)	0R (95% CI)	0R (95% CI)	
Boys				
No. of With/without wheezing	81/1035	113/1189	97/899	
Crude	1.00	1.21 (0.90–1.63)	1.37 (1.01–1.87)	**0.04**
Model 1	1.00	1.20 (0.89–1.62)	1.31 (0.96–1.78)	0.08
Model 2	1.00	1.14 (0.84–1.54)	1.13 (0.82–1.55)	0.45
Model 3	1.00	1.14 (0.84–1.54)	1.13 (0.82–1.55)	0.45
Girls				
No. of With/without wheezing	112/1499	108/1366	98/1070	
Crude	1.00	1.05 (0.8–1.39)	1.22 (0.92–1.62)	0.16
Model 1	1.00	1.05 (0.79–1.38)	1.19 (0.89–1.58)	0.22
Model 2	1.00	0.99 (0.75–1.30)	1.04 (0.78–1.39)	0.76
Model 3	1.00	0.99 (0.75–1.30)	1.04 (0.78–1.40)	0.75
Whole population				
No. of With/without wheezing	193/2534	221/2555	195/1969	
Crude	1.00	1.13 (0.92–1.38)	1.30 (1.05–1.60)	**0.01**
Model 1	1.00	1.11 (0.91–1.36)	1.24 (1.00–1.53)	**0.04**
Model 2	1.00	1.05 (0.86–1.29)	1.08 (0.87–1.33)	0.46
Model 3	1.00	1.05 (0.86–1.29)	1.08 (0.87–1.34)	0.45

Model 1: adjusted for age and sex (for total participants).

Model 2: further adjusted for watch TV and computer use.

Model 3: additionally adjustment for BMI.

Significant values are in [bold].

## Discussion

In this study we found that participants with higher adherence to western pattern were more likely to have wheezing in the past 12 months in boys and in the whole populations.

We found a significant positive association between a western dietary pattern and wheezing in the past 12 months among whole participants and boys, but after adjustment for confounder this association remained significant only in boys. Consistent with our results, a cross-sectional study on children and adolescents showed that there is a significant relationship between western diet and wheezing^33^. Moreover, McKeever et al.^34^ combined cross-sectional and longitudinal studies and they found an increasing trend in association between refined diet pattern, which was similar to western diet, and wheeze prevalence. Also, studies have shown that consuming fast foods, which are an inseparable part of western diets, is correlated with asthma and wheezing^35,36^. In contrast with our results a study illustrated that there is an inverse relationship between maternal western dietary pattern and presence of wheeze in Japanese toddlers^37^. However, the author of this article stated that the Western diet common in Japan may be healthier than the Western diet used in countries such as the United States^37^, and this could explain the inconsistency between the results. Furthermore, a systematic review and meta-analysis failed to show any association between dietary patterns, including the Western diet, and asthma and wheezing^38^.

There is not much research on the relationship between asthma symptoms and the western diet. But the available evidence shows that since the western diet is obesogenic, it can worsen asthma conditions^39^. Obesity can have adverse effects on asthma in many ways such as reducing lung volume^40^ and function^41^ and also worsening asthma symptoms like wheezing^42^. On the other hand, the western dietary pattern which has a high omega-6 fatty acids, can affect asthma symptoms through inflammatory pathways^43^. Arachidonic acid which is produced through omega-6 fatty acids metabolism can be converted into inflammatory mediators such as leukotrienes and prostaglandins^44^. Furthermore, western diet is low in fruits, vegetables and whole grains^19^ which causes the low antioxidant content of this diet. Individuals with asthma are more sensitive to triggers such as allergens, and contact with these triggers causes inflammation and release of ROS, therefore, they are more exposed to oxidative stress^45^. Also, the lack of antioxidants and the release of ROS can activate the nuclear factor-kB, which resulted in increased production of inflammatory cytokines^46^. In addition, recent studies have revealed that gut microbiota can affect asthma through western diet intake^47^. Food intake affects the composition of gut bacteria and consequently the metabolism of nutrients^48^. For example, an animal study demonstrated that consuming a high fiber diet-unlike western diet- by changing the gut microbiota, increases short-chain fatty acids levels and, as a result, reduces inflammation^49^.

In our study no relation was found between adherence to western dietary pattern and wheezing in the past 12 months in the girls’ population. Some evidence has shown that the incidence of asthma and wheezing in childhood is higher in boys than in girls^50^. This difference can be due to the smaller size of airways in boys, which has caused greater sensitivity to allergens^51^. Maybe it can explain the reason for not finding an association in girls.

In the present research no significant association was found between adherence to western dietary pattern and current asthma, asthma confirmed by a doctor and use of asthma medication. But also our results showed a significant negative trend in the relation between western diet and current asthma in boys. The previous results in this matter are inconsistent. Some studies resulted that a strong adherence to western diet can lead to a higher risk of current asthma in children^52^, however some other, the same as our study, could not find any significant association^18^.

This is the first study that examines the association between western dietary pattern and asthma with a large sample size in the Middle East. Our large sample size is a good representative of the researched population. Also, subgroup analyzes were performed based on sex. In addition, a valid questionnaire was used to collect the participants' information. However, this study also has some limitations that are mentioned. The grams of participants’ food intake could not be calculated by our questionnaire and it may affect our findings. Furthermore, using the food frequency questionnaire can lead to memory and reporting bias. In addition, the design of our study was cross-sectional and it is well known that a causal relationship cannot be obtained in this design. However, it should be mentioned that although in our study a causal relationship was not found, we have assumed that adherence to the western dietary pattern can lead to an increase in the risk of wheezing in asthma sufferers. This result can be a light for future research.

In summary, the adherence to western dietary pattern may be related to higher risk of wheezing in asthmatic children. However, no association was found between western diet and asthma confirmed by a doctor, current asthma and use of asthma medication. But asthma sufferers should take caution in receiving such unhealthy diets. More and stronger studies are needed for better conclusions.

## Data Availability

The datasets generated and/or analysed during the current study are available from the corresponding author on reasonable request.
